# The Modulation of Phospho-Extracellular Signal-Regulated Kinase and Phospho-Protein Kinase B Signaling Pathways plus Activity of Macrophage-Stimulating Protein Contribute to the Protective Effect of Stachydrine on Acetaminophen-Induced Liver Injury

**DOI:** 10.3390/ijms25031484

**Published:** 2024-01-25

**Authors:** Fu-Chao Liu, Huang-Ping Yu, Hung-Chen Lee, Chun-Yu Chen, Chia-Chih Liao

**Affiliations:** 1 Department of Anesthesiology, Chang Gung Memorial Hospital, Taoyuan 333, Taiwan; ana5189@cgmh.org.tw (F.-C.L.); yuhp2001@cgmh.org.tw (H.-P.Y.); m7079@cgmh.org.tw (H.-C.L.); an5376@cgmh.org.tw (C.-Y.C.); 2 College of Medicine, Chang Gung University, Taoyuan 333, Taiwan

**Keywords:** stachydrine, acetaminophen, liver injury, inflammation, oxidative stress, ERK, AKT, macrophage-stimulating protein

## Abstract

Stachydrine, a prominent bioactive alkaloid derived from Leonurus heterophyllus, is a significant herb in traditional medicine. It has been noted for its anti-inflammatory and antioxidant characteristics. Consequently, we conducted a study of its hepatoprotective effect and the fundamental mechanisms involved in acetaminophen (APAP)-induced liver injury, utilizing a mouse model. Mice were intraperitoneally administered a hepatotoxic dose of APAP (300 mg/kg). Thirty minutes after APAP administration, mice were treated with different concentrations of stachydrine (0, 2.5, 5, and 10 mg/kg). Animals were sacrificed 16 h after APAP injection for serum and liver tissue assays. APAP overdose significantly elevated the serum alanine transferase levels, hepatic pro-inflammatory cytokines, malondialdehyde activity, phospho-extracellular signal-regulated kinase (ERK), phospho-protein kinase B (AKT), and macrophage-stimulating protein expression. Stachydrine treatment significantly decreased these parameters in mice with APAP-induced liver damage. Our results suggest that stachydrine may be a promising beneficial target in the prevention of APAP-induced liver damage through attenuation of the inflammatory response, inhibition of the ERK and AKT pathways, and expression of macrophage-stimulating proteins.

## 1. Introduction

Drug-induced liver damage poses a serious risk to human health and can potentially result in acute liver failure. Acetaminophen (APAP) is a commonly used antipyretic and analgesic medication in clinical settings [1]. Upon overdose, APAP is metabolized to excess N-acetyl-p-benzoquinone imine (NAPQI), a toxic reactive intermediate, via the cytochrome P-450 system [2]. NAPQI is eliminated by primary enzymatic antioxidant defense mechanisms within cellular storage, including glutathione (GSH) and superoxide dismutase (SOD) [3]. Nevertheless, the excessive production of NAPQI depletes these protective enzymes, causing compromised mitochondrial activity and the production of reactive oxygen species (ROS), ultimately leading to DNA damage and hepatocyte death [4].

Toxic APAP metabolites induce early liver damage, and subsequent innate immunity and downstream inflammatory mediators exacerbate the injury. Following initial injury to hepatocytes, resident phagocytic macrophages are stimulated by damage-associated molecular pattern (DAMP) molecules, including nuclear and mitochondrial DNA fragments [5]. These activated macrophages subsequently secrete pro-inflammatory chemokines and cytokines, including tumor necrosis factor (TNF)-α, interleukin (IL)-1β, IL-6, and ROS to attract more immune cells into the liver vasculature. This exacerbates the damage caused by APAP-induced liver injury [6].

Macrophage-stimulating protein (MSP) is a circulating serum protein, alternatively recognized as hepatocyte growth factor-like protein, which plays a role in inflammation and immune regulation. It is synthesized and expressed in hepatic parenchymal cells. Evidence suggests that MSP exerts its biological actions through the activation of the receptor tyrosine kinase known as recepteur d’origine nantais (RON), and targets macrophages and other cell types [7,8]. This MSP function is important for immune response against infection and inflammation. It induces chemotactic responses, facilitates migration, enhances macrophage phagocytosis, and promotes the dispersion of resident macrophages [9]. A study found that MSP can induce macrophages to release inflammatory cytokines and produce oxidative stress during smoke-induced airway inflammation [10].

Recent research findings have indicated that the extracellular signal-regulated kinase (ERK), belonging to the mitogen-activated protein kinase (MAPK) family, is involved in the modulation of oxidative stress and inflammation [11]. Attenuation of the ERK signaling pathway decreases the inflammatory process during APAP-induced hepatic injury [12]. Furthermore, recent research has highlighted the significant involvement of protein kinase B (AKT) in the signaling pathway that limits inflammatory responses following injury. Additionally, AKT demonstrates protective effects against acute liver damage induced by APAP [13]. In a separate investigation, it has demonstrated that the pro-inflammatory response of liver-resident macrophages mediates the expansion of APAP-induced acute liver damage via the AKT pathway. Activation of AKT proves vital in regulating macrophage phagocytosis and inflammatory cytokine production [14].

Stachydrine (N,N-dimethyl-L-proline) (ST) is a major bioactive alkaloid extracted from Leonurus heterophyllus, an significant botanical remedy used in traditional medicine. Previous analysis study showed that the composition of ST in the plant is about 0.5–1.5% [15]. It presents various biological characteristics, including anti-inflammatory and antioxidant properties [16,17]. Previous evidence has demonstrated that ST inhibits excessive autophagy by reducing ROS production and inhibiting NADPH oxidase 2 activity [18]. Another study confirmed that ST provides protection against liver fibrosis induced by CCl_4_ by suppressing pathways associated with inflammation and oxidative stress [19]. These studies indicate that ST holds significance in both inflammatory responses and oxidative stress. However, its pharmacological effect in APAP-induced liver damage remains unexplored. Therefore, the objective of this study was to assess the mechanism of ST in response to liver injury induced by APAP in a mouse model.

## 2. Results

### 2.1. Effects of ST on HepG2 Cell Viability

**Figure 1:**
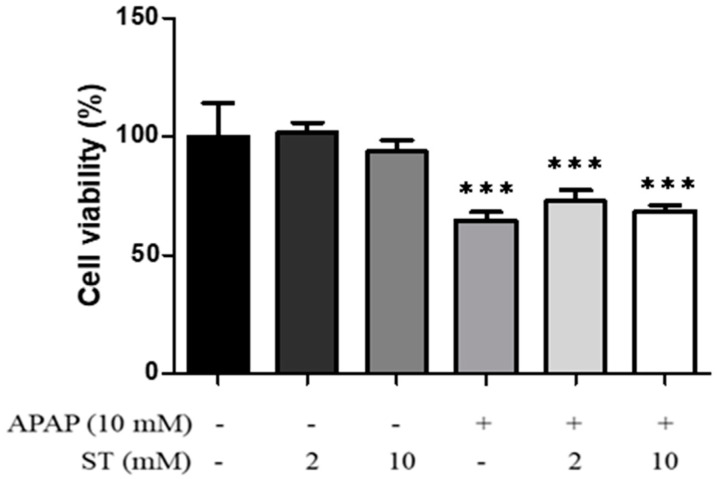
HepG2 cells were treated with varying concentrations of ST (0, 2, and 10 mM) for a duration of 24 h. The outcomes were depicted as a percentage relative to the control and represented as the mean ± SEM. *** *p* < 0.005 compared to the control group.

We first investigated the impacts of increasing ST concentrations on the viability of HepG2 cells following a 24 h treatment. As illustrated in Figure 1, exposure to ST (2 and 10 mM) did not lead to a significant alteration in cell viability. In contrast, the administration of APAP (10 mM) substantially reduced cell viability compared to the control group (*p* < 0.005). However, the presence of ST (2 and 10 mM) did not yield a significant effect on the viability of HepG2 cells in conjunction with APAP. 

### 2.2. Effects of ST on the Expression of ERK and AKT in HepG2 Cells

**Figure 2:**
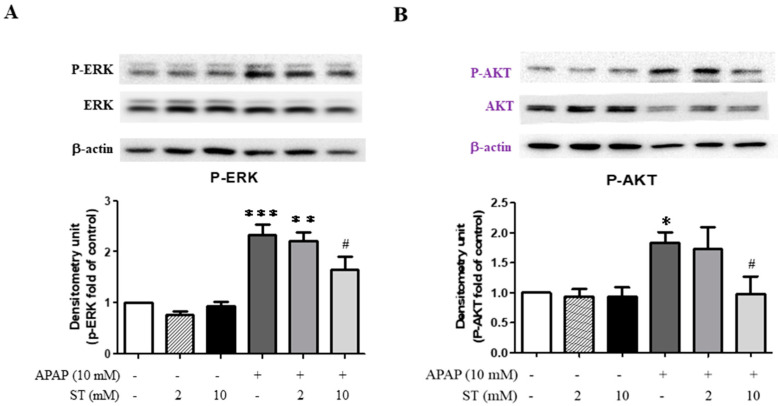
Effects of ST treatment on APAP-stimulated ERK and AKT expression in HepG2 Cells. The protein expressions of ERK and AKT were analyzed by Western blot, respectively. (**A**) ERK protein expression level. (**B**) AKT protein expression level. β-actin was used as the protein loading control. Band intensities were assessed via densitometry, and each value is presented as the mean ± SEM. * *p* < 0.05, ** *p* < 0.01, *** *p* < 0.005 compared to the control group; # *p* < 0.05 compared to the APAP-alone-treated group.

Furthermore, the expression of P-ERK and P-AKT proteins was significantly enhanced in APAP-treated HepG2 cells compared to that in the control group (*p* < 0.005 and <0.05, respectively) (Figure 2A,B). Treatment with a lower ST dose (2 mM) did not significantly reverse the ERK and AKT phosphorylation compared to the APAP-alone-treated group. However, after treatment with a higher ST dose (10 mM), the increased expression of P-ERK and P-AKT proteins induced by APAP was inhibited compared to that of the APAP-alone-treated group (*p* < 0.05). 

### 2.3. ST Ameliorated APAP-Induced Hepatic Injury

**Figure 3:**
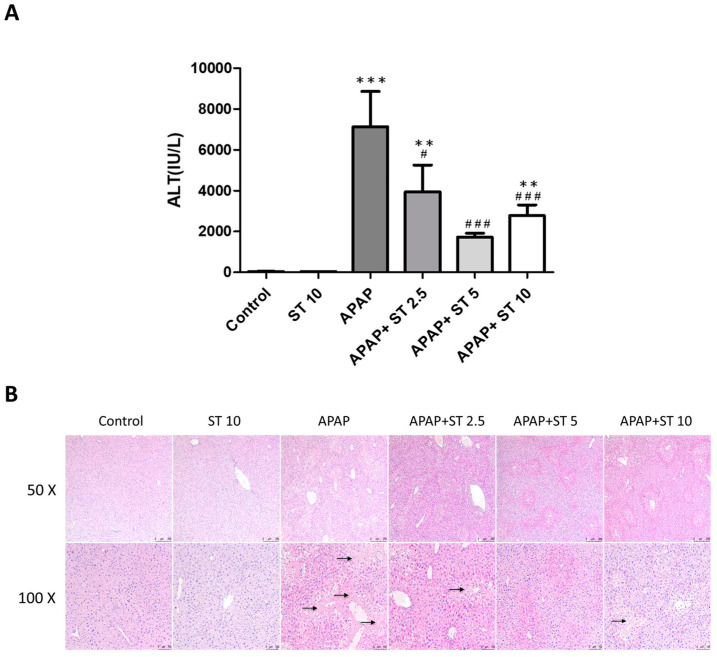
Effects of ST treatment in APAP-induced liver injury. Mice received saline (control), hepatotoxic injection of APAP (300 mg/kg), or different concentrations of ST (2.5, 5, and 10 mg/kg) 30 min after APAP administration. Serum was collected 16 h after APAP administration. (**A**) The serum ALT data are presented as means ± SEM. ** *p* < 0.01, *** *p* < 0.005 compared to the control group; # *p* < 0.05, ### *p* < 0.005 compared to the APAP group. (**B**) Hematoxylin and eosin (H&E) staining was conducted on liver tissues from six groups. Representative images were selected from each group. Black arrows indicate hepatocyte necrosis.

Liver ALT levels were elevated after a single APAP toxic dose (300 mg/kg) (*p* < 0.005) compared to those in the normal group (Figure 3A). After 30 min of APAP administration, ST significantly reduced serum ALT levels. Serum ALT levels were markedly lower in the APAP combined with ST groups (5 and 10 mg/kg) than those in the APAP-only group (*p* < 0.005). Histopathological analysis revealed sinusoidal congestion and centrilobular necrosis of the liver parenchyma in the APAP group (Figure 3B). In line with the findings regarding serum ALT levels, ST treatment significantly decreased pathological features following APAP-induced liver injury, resulting in less necrosis and well-preserved liver parenchyma. 

### 2.4. Effect of ST on Macrophage Accumulation in APAP-Induced Hepatic Injury

**Figure 4:**
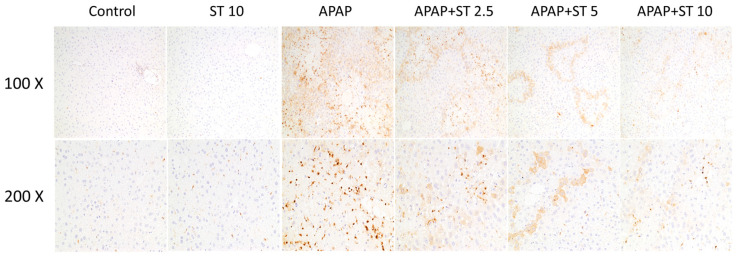
Effects of ST treatment on macrophage infiltrations in APAP-induced liver injury. Mice received saline (control), hepatotoxic injection of APAP (300 mg/kg), or different concentrations of ST (2.5, 5, and 10 mg/kg) 30 min after APAP injection. All mice were sacrificed 16 h after treatment for analysis by immunohistochemistry. Liver tissues were immunostained with Mac-2 antibody (brown) from 6 groups. Representative images were selected from each group.

In order to assess macrophage infiltration subsequent to APAP-induced hepatic injury, liver tissue underwent immunohistochemically staining using Mac-2, a specific antibody targeting macrophages. In the APAP group, there was evident macrophage infiltration around the injured region within the liver parenchyma, as compared to the control group (Figure 4). The groups receiving ST treatment after APAP administration displayed a marked reduction in macrophage accumulation compared to animals treated solely with APAP.

### 2.5. Influence of ST on the Levels of Inflammatory Cytokine in Liver Tissues

**Figure 5:**
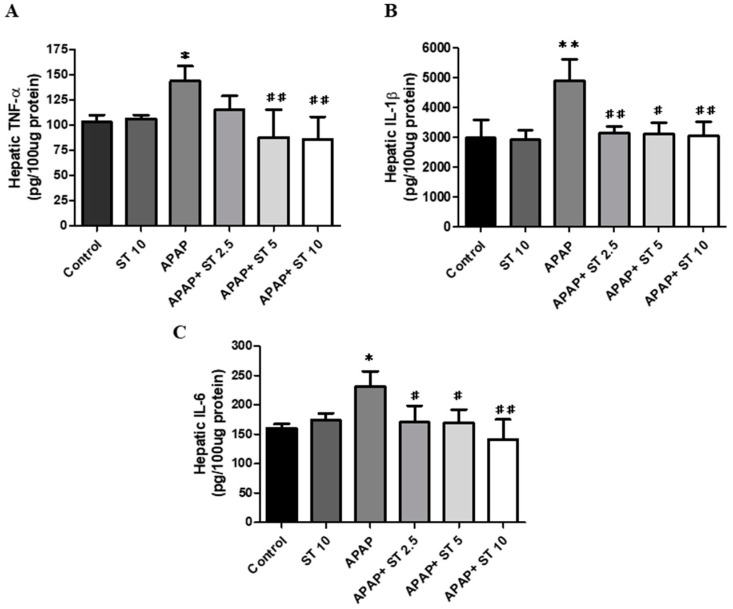
Effects of ST treatment on hepatic cytokine levels of (**A**) TNF-α, (**B**) IL-1β, and (**C**) IL-6 in APAP-induced liver injury. Mice received saline (control), hepatotoxic injection of APAP (300 mg/kg), or different concentrations of ST (2.5, 5, and 10 mg/kg) 30 min after APAP injection. All mice were sacrificed 16 h after treatment for analysis. Each value is presented as mean ± SEM. * *p* < 0.05, ** *p* < 0.01 compared to the control group; # *p* < 0.05, ## *p* < 0.01 compared to the APAP group.

To evaluate pro-inflammatory cytokines expression following APAP-induced hepatic injury, we measured the levels of TNF-α, IL-1β, and IL-6 in liver tissues. As shown in Figure 5, 16 h after APAP injection, these cytokines were significantly increased compared to those in the control group. After APAP administration for 30 min, ST (2.5 mg/kg) significantly decreased hepatic IL-1β and IL-6 levels (*p* < 0.01 and <0.05, respectively), but not significant difference was observed in TNF-α levels compared with those of the APAP-only treated animals. In addition, treatment with a higher ST dose (5 and 10 mg/kg) markedly reduced TNF-α, IL-1β, and IL-6 levels.

### 2.6. ST Decreased APAP-Induced Oxidative Stress Injury 

**Figure 6:**
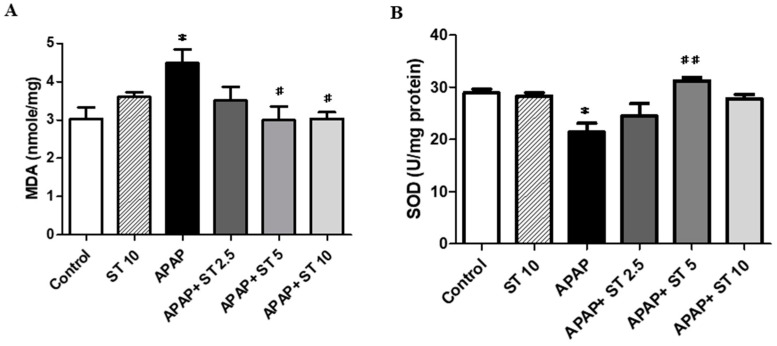
Effects of ST treatment on MDA and SOD activities in APAP-induced liver injury. Mice received saline (control), hepatotoxic injection of APAP (300 mg/kg), or different concentrations of ST (2.5, 5, and 10 mg/kg) 30 min after APAP injection. All mice were sacrificed, and liver tissues were measured 16 h after treatment. (**A**) Mean ± SEM values are shown for MDA, and (**B**) for SOD levels. * *p* < 0.05 compared to the control group; # *p* < 0.05, ## *p* < 0.01 compared to the APAP group.

APAP overdose induced oxidative stress injury and activated antioxidant defense system. We measured MDA and SOD levels as indicators of oxidative stress injury in liver tissues. The MDA concentration in the APAP group was markedly elevated compared to the normal group (*p* < 0.05) (Figure 6A). After ST treatment (5 and 10 mg/kg), it markedly decreased compared to that in APAP-treated mice (*p* < 0.05). Moreover, excessive oxidative stress decreases the activity of SOD, an important antioxidant enzyme. The SOD levels in the APAP group were notably lower compared to those in the normal group (*p* < 0.05) (Figure 6B). However, hepatic SOD activity was restored after treatment with 5 mg/kg ST (*p* < 0.01).

### 2.7. Effect of ST on ERK and AKT Expression in Liver Tissues

**Figure 7:**
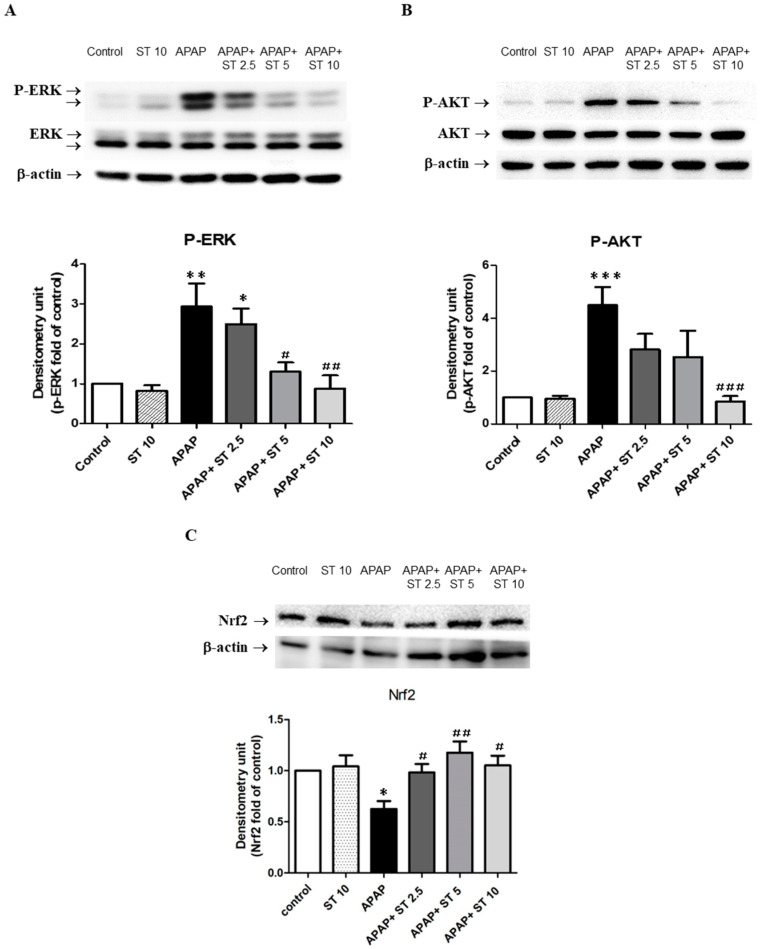
Effects of ST treatment on hepatic (**A**) ERK, (**B**) AKT, and (**C**) Nrf2 expressions. Mice received saline (control), hepatotoxic injection of APAP (300 mg/kg), or different concentrations of ST (2.5, 5, and 10 mg/kg) 30 min after APAP injection. All mice were sacrificed 16 h after treatment for Western blot analysis of liver ERK, AKT, and Nrf2 expression levels. Band intensities were assessed via densitometry, and each value is presented as mean ± SEM. * *p* < 0.05, ** *p* < 0.01, *** *p* < 0.005 compared to the control group; # *p* < 0.05, ## *p* < 0.01, ### *p* < 0.005 compared to the APAP group.

We investigated the expressions and phosphorylation of hepatic ERK and AKT following APAP-induced hepatic injury. Western blot analysis showed that phospho-ERK and phospho-AKT expressions significantly increased in a single exposure to APAP compared with that in the control group (*p* < 0.01 and <0.005, respectively) (Figure 7A,B). Treatment with a low ST dose (2.5 mg/kg) 30 min after APAP administration showed no significant differences in phospho-ERK and phospho-AKT levels compared to those in the APAP-only group. However, treatment with a higher ST dose (10 mg/kg) markedly reduced phospho-ERK and phospho-AKT expression compared with that in the APAP group (*p* < 0.01 and <0.005, respectively).

### 2.8. Effect of ST on Nrf2 Expression in Liver Tissues

We also investigated the expressions of Nrf2 in APAP-induced hepatotoxicity. Western blot analysis unveiled that the expression of Nrf2 was significantly decreased in a single exposure to APAP compared with that in the control group (*p* < 0.05) (Figure 7C). ST treatment (2.5, 5, and 10 mg/kg) had significantly increased Nrf2 expression levels compared to the APAP group (*p* < 0.05 and <0.01). The results showed that the protective effect of ST in APAP-induced liver injury was related to upregulation of Nrf2 expression.

### 2.9. Effect of ST on MSP Expression in Liver Tissues 

**Figure 8:**
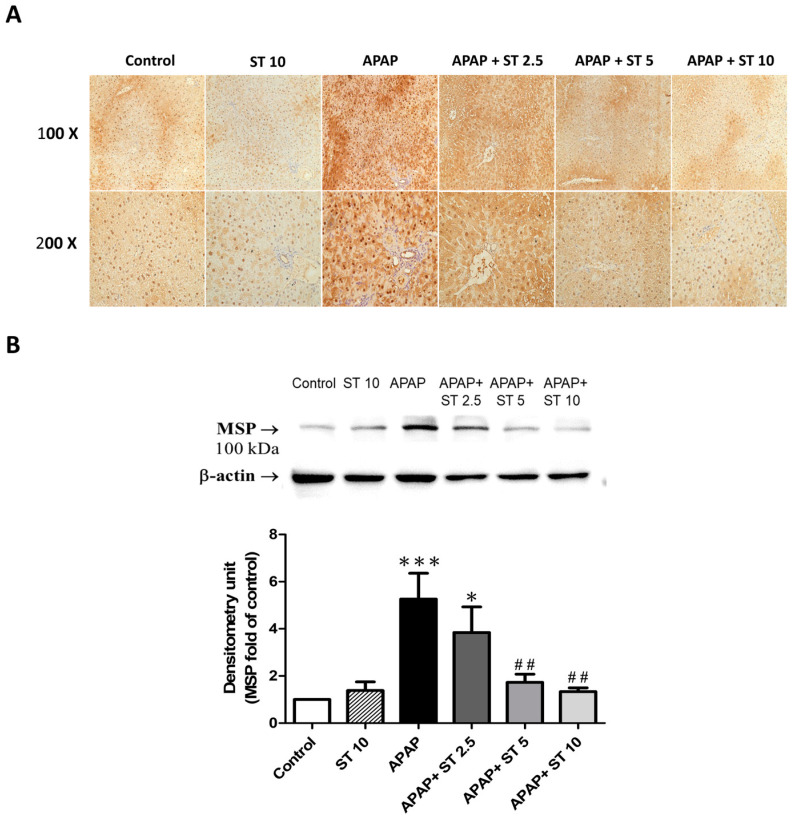
Effects of ST treatment on MSP expression in APAP-induced liver injury. Mice received saline (control), hepatotoxic injection of APAP (300 mg/kg), or different concentrations of ST (2.5, 5, and 10 mg/kg) 30 min after APAP injection. All mice were sacrificed, and liver tissues were collected 16 h after treatment. (**A**) Immunohistochemical staining illustrating liver MSP expression (brown) across the six groups. Representative images were selected from each group. (**B**) Liver MSP expression levels. Band intensities were assessed via densitometry, and each value is presented as mean ± SEM. * *p* < 0.05, *** *p* < 0.005 compared to the control group; ## *p* < 0.01 compared to the APAP group.

In order to explore the potential anti-inflammatory mechanism of ST in response to APAP-induced hepatic injury, we conducted immunohistochemical staining and Western blot analyses utilizing an MSP antibody. The APAP-only group showed increased hepatic MSP expression in liver tissues compared to those in the control group (Figure 8A). The groups treated with ST (5 and 10 mg/kg) exhibited notably reduced MSP expression in the liver parenchyma. In addition, MSP levels significantly increased after APAP administration compared to those in the control group (*p* < 0.005) (Figure 8B). Notably, ST treatment (5 and 10 mg/kg) significantly reduced the hepatic MSP levels after APAP challenge (*p* < 0.01 both). 

## 3. Discussion

In the present study, we found that ST, a major bioactive alkaloid purified from Leonurus heterophyllus, decreased post-treatment APAP-induced hepatotoxicity, inflammation, and oxidation in a mouse model. ST exhibited a notable decrease in inflammatory reactions, including histopathological changes, infiltration of macrophages, and the release of pro-inflammatory cytokines. In addition, ST reduced the expression of hepatic phospho-ERK, phospho-AKT, and MSP.

An APAP overdose causes severe hepatotoxicity and acute liver failure. In this study, we administered an intraperitoneal dose of 300 mg/kg APAP to induce acute liver damage. Excess production of the metabolite NAPQI leads to hepatocyte damage, and subsequent innate immune cell recruitment and activation contribute to the amplification of APAP-induced acute liver injury [20]. After the initial hepatocyte apoptosis or necrosis, the activation of hepatic macrophages is induced by DAMP molecules through recognition by Toll-like receptors [21]. Activated resident macrophages release inflammatory cytokines, including TNF-α, IL-1β, and IL-6, to recruit infiltrating macrophages and neutrophils into areas of necrosis that contribute to the subsequent severe liver damage [22]. The roles of resident liver macrophages as a first-line defense against the innate immune system has been shown in some liver injury models [23]. Previous studies have shown that ST exerts anti-inflammatory effects in CCl_4_-induced liver fibrosis [19]. Consistent with our results, the administration of ST following APAP-induced injury effectively diminished both macrophage accumulation and the expression of inflammatory cytokines. These results suggest a protective role for ST in ameliorating the progression and severity of inflammation after APAP-induced liver injury. 

APAP-induced hepatotoxicity is characterized by oxidative stress. During APAP overdose, excessive NAPQI consumes protective antioxidant enzymes, resulting in the overproduction of ROS and free radicals [4]. A recent study reported that ST exerts hepatoprotective effects by inhibiting oxidative stress in CCl_4_-induced liver fibrosis [19]. Another study reported that ST treatment attenuates oxidative stress in a rat model of cardiac hypertrophy [16]. Our results showed that ST treatment significantly decreased MDA levels after APAP overdose, indicating reduced ROS production. In addition, treatment with ST elevated the activity of antioxidant enzymes such as SOD. These findings indicate that ST contributes to decreased oxidative stress following APAP-induced hepatotoxicity. 

MSP and its specific receptor target macrophages to regulate their motility and phagocytic activity [24]. Recent evidence has indicated a correlation between MSP and inflammation. MSP and its specific receptor may exert a synergistic effect to activate macrophages and increase oxidative stress and cytokine production during smoke-induced airway inflammation in rats [10]. Previous studies have demonstrated that MSP treatment upregulated the expression of pro-inflammatory and apoptotic genes in the liver during early changes in hepatic inflammation [25]. Previous studies have indicated that knockout of the Ron receptor tyrosine kinase domain protects against endotoxin-induced liver damage [26]. In this study, we investigated the effect of MSP in combination with ST against APAP-induced hepatotoxicity. Our results showed that the APAP challenge increased the expression of MSP, and ST reduced the levels of MSP expression in APAP-induced liver injury. However, the MSP-RON signaling pathway seems to exert discriminating effects and may act as an anti-inflammatory mediator during inflammatory reactions through contradictory mechanisms. Previous studies have indicated that MSP inhibits the expression of pro-inflammatory cytokines in HepG2 cells and acts as a negative regulator of inflammation in a non-alcoholic steatohepatitis model [27]. The activation of the MSP-RON pathway diminishes the production of inflammatory cytokines induced by LPS, and the removal of RON receptors impairs the anti-inflammatory capacity of the liver following LPS stimulation [28]. Another study revealed both pro- and anti-inflammatory effects in human alveolar macrophages by engaging the MSP-RON pathway [29]. These results suggest that the effects of MSP on inflammation are complex. MSP may participate in different pathological signaling processes between cells. Further investigations are required to clarify the modulation of MSP activity in acute and chronic inflammatory states.

The initiation of intracellular signaling pathways can further evoke both inflammatory reactions and oxidative stress. Among these, MAPK family members are critical signaling components involved in oxidative stress and inflammation [30]. Previous studies have demonstrated that oxidative stress can trigger the activation of MAPK, including ERK, during APAP-induced liver damage [31]. It is conceivable that transition of ROS may be required for an ERK signaling pathway activation [11]. The protective effects observed in APAP-induced hepatotoxicity are associated with the inhibition of the ERK pathway and reduced oxidative stress [32,33]. A recent study reported that ST could induces apoptosis and inhibits ERK proteins in different cell types [34]. In our study, based on Western blot results from cellular and animal models, phospho-ERK protein expression significantly increased after APAP challenge. High ST doses effectively decreased this phosphorylation, suggesting protective effects against APAP toxicity through the ERK signaling pathway. 

In addition, AKT, a vital signaling pathway, participates in numerous cellular processes, including inflammatory responses and cell survival to injury [35]. Previous studies have revealed that the AKT signaling pathway can negatively regulate inflammatory responses and decrease mortality following sepsis and ischemia/reperfusion injury [36]. Additionally, recent studies have demonstrated that inhibiting the AKT signaling pathway leads to a reduction in lipopolysaccharide-induced inflammation. This reduction is achieved by decreasing cytokine expression, lowering ROS production, and inhibiting the polarization of M1 macrophages [37,38]. A previous study reported that inhibition of AKT-mediated ROS production in liver-resident macrophages reduced APAP-induced liver injury in a hyperglycemic mouse model [14]. Another study showed that ST protects against traumatic brain injury form neuronal injury in rats by attenuating PI3K/AKT pathway expression [39]. In our study, we found that ST significantly decreased AKT phosphorylation in liver tissues. Our results indicate beneficial effect of ST on the AKT pathway involved in liver injury.

APAP toxicity induces the formation of ROS and compromises the antioxidant capacity. Nrf2, a pivotal transcription factor and a key regulator of oxidative stress, performs crucial functions to regulate the expression of antioxidant genes, thereby providing protection against cell injury. Nrf2, typically located in an inactive state within the cytoplasm, undergoes activation upon exposure to oxidative stress and, subsequently, it can translocate from the cytoplasm to the nucleus, where it binds to the antioxidant response element, initiating the antioxidation, including SOD and heme oxygenase-1 (HO-1) [40]. Earlier research indicates that Nrf2-knockout mice exhibit more severe APAP-induced liver injury compared to their wild-type. The mechanism against APAP-induced hepatotoxicity is associated with the activation of Nrf2 and the reduction in liver oxidative stress [41]. In our study, we investigated the mechanism of Nrf2 in ST against oxidative stress in APAP-induced hepatotoxicity. Previous reports have demonstrated that ST ameliorates hypoxia reoxygenation injury in cardiomyocytes by activation of the Nrf2 pathway [42]. In this study, we demonstrated that ST induces an upregulation in Nrf2 expression, promoting its translocation into the nucleus. These findings imply that the activation of Nrf2 protein by ST potentially plays a role in protective effects against APAP-induced liver injury.

In conclusion, our study showed that the administration of ST effectively mitigates APAP-induced hepatotoxicity in a mouse model. Its antioxidant and anti-inflammatory mechanisms involve the inhibition of the ERK and AKT pathways, along with the downregulation of the expression of macrophage- stimulating proteins. Therefore, ST is a promising beneficial target for APAP-induced liver damage. Additional investigations are required to confirm its prospective application.

## 4. Materials and Methods

### 4.1. Animals 

Male C57BL/6 mice (10–12 weeks of age) were purchased from BioLASCO Taiwan Co., Ltd. (Taipei, Taiwan). All protocols involving animals were reviewed and approved by the Institutional Animal Care and Use Committee of Chang Gung Memorial Hospital. The animal experiments were conducted in strict accordance with the ethical principles outlined in the Animal Welfare Act and the Guide for the Care and Use of Laboratory Animals issued by the National Institutes of Health. The animals were housed in environments with controlled conditions and were maintained under a 12 h light/12 h dark cycle at the Laboratory Animal Center of Chang Gung Memorial Hospital. The animals underwent an overnight fasting period prior to the experiments.

### 4.2. Cell Culture

The human hepatocellular carcinoma (HepG2) cell line was procured from American Tissue Culture Collection (ATCC, Rockville, MD, USA). These cells were cultured in high-glucose Dulbecco’s Modified Eagle’s Medium supplemented with 10% fetal bovine serum and 1% antibiotic-antimycotic solution (#15240062, Gibco, Grand Island, NY, USA). They were maintained in a 5% CO_2_ incubator at 37 °C, with the culture medium refreshed every 48 h. 

### 4.3. Cell Viability Assay

Cell viability was assessed utilizing the Cell Counting kit-8 (CCK-8). Cells were removed from the culture dish with 0.25% trypsin, diluted to a final density of 1 × 105 cells/mL in fresh medium, and 100 μL (1 × 104 cells/well) of the cell suspension was seeded in 96-well plates. After adherence to the bottom of the plates, cells were treated with different doses of ST (2 and 10 mM) (Cayman Chemical Co., Ann Arbor, MI, USA) over a 24 h duration. Following incubation, each well was replaced with 100 μL fresh medium and treated with and without 10 mM APAP. Following a 24 h period, 10 μL of the CCK-8 solution was introduced into each well and allowed to incubate for 2 to 4 h. Afterward, the absorbance of the contents in each well was gauged at 450 nm. Cell viability is quantified as a percentage relative to untreated control cells. 

### 4.4. Experimental Design

Mice were assigned to six groups randomly, with each group consisting of six mice: control (saline), ST only (10 mg/kg), APAP only (300 mg/kg), APAP + ST (2.5 mg/kg), APAP + ST (5 mg/kg), and APAP + ST (10 mg/kg). The experimental group was administered a hepatotoxic intraperitoneal dose of 300 mg/kg APAP (Sigma Chemical Co., St. Louis, MO, USA) dissolved in warm normal saline (0.9%) to achieve a concentration of 20 mg/mL, while the control group was administered an equal volume of standard saline solution. Thirty minutes after APAP administration, the mice were intraperitoneally injected ST at doses of 0, 2.5, 5, or 10 mg/kg. Sixteen hours following the administration of APAP, the animals were humanely euthanized via cervical dislocation under isoflurane anesthesia. Blood samples were then obtained from the vena cava and subsequently subjected to centrifugation for the assessment of liver enzyme activity. Immediately after collection, liver tissues were harvested for subsequent analyses.

### 4.5. Serum Enzyme Determination

Serum alanine aminotransferase (ALT) levels were measured to evaluate hepatic injury. Blood samples were gathered and then subjected to centrifugation centrifuged at 12,000× *g* for 10 min. The resulting serum was utilized for liver function testing using a Vitros DT60 II Chemistry System (Ortho-Clinical Diagnostics, Johnson & Johnson, New York, NY, USA). All procedures related to the processing of serum samples strictly followed the instructions provided by the manufacturer. 

### 4.6. Histology Analysis of Liver Tissues

Liver tissues were collected, fixed in a 4% paraformaldehyde solution in PBS (pH 7.4) for 24 h, embedded in paraffin, and then cut into 4 μm thick sections. Hepatic slices were subjected to conventional hematoxylin and eosin (H&E) staining and subsequently examined for signs of liver damage using a DM2500 light microscope (Leica, Wetzlar, Germany). 

### 4.7. Immunohistochemical Analysis on Liver Tissues

Liver sections were dewaxed followed by a 30 min blocking step using a blocking buffer and incubated with anti-Mac and anti-MSP antibodies (BD Biosciences Pharmingen, San Diego, CA, USA) at 37 °C for 2 h. After the incubation period, the segments were washed with PBS for a duration of 5 min, followed by exposure to biotin–horseradish and streptavidin–horseradish peroxidase-conjugated secondary antibodies for one hour. Subsequently, the slides were stained with DAB and counterstained with hematoxylin in accordance with the manufacturer’s guidelines (Millipore IHC select kit; Burlington, MA, USA). The incubation times for all samples were consistent. Positive staining, characterized by a cytoplasmic or nuclear brownish-yellow color in the liver cells, was evaluated through a light microscope. 

### 4.8. Measurement of Cytokine Levels in Liver Tissues

The concentrations of TNF-α, IL-1β, and IL-6 in hepatic tissues were quantified using ELISA kits from eBiosciences (San Diego, CA, USA) in according with the manufacturer’s guidelines. In summary, Hepatic tissues were initially homogenized on ice and subsequently centrifuged at 12,000× *g* for 10 min at 4 °C. The resulting supernatants were then placed into a 96-well plate pre-coated with antibodies specific to TNF-α, IL-1β, and IL-6. This was followed by the addition of an HRP-conjugated streptavidin solution. The absorbance of each target cytokine was measured at 450 nm. Subsequently, the levels of TNF-α, IL-1β, and IL-6 were normalized based on the tissue weight.

### 4.9. Measurement of Liver Oxidative Stress Markers

The liver tissue samples were homogenized in 10% trichloracetic acid and centrifuged at 1000× *g* at 4 °C for 15 min. The supernatant was removed and subsequently subjected to re-centrifuged at 35,000× *g* at 4 °C for 8 min. The final supernatant was utilized for evaluating levels of malondialdehyde (MDA) and superoxide dismutase (SOD). The measurement of lipid peroxidation, represented by MDA was conducted using a Bioxytech MDA-586 kit (OxisResearch, Portland, OR, USA). Meanwhile, SOD activity was quantified using spectrophotometric analysis. All protocols were conducted in accordance with the manufacturer’s provided instructions. 

### 4.10. Western Blot Analysis

Liver tissues were initially lysed in a commercial buffer, and the resulting cell lysates were centrifuged at 12,000× *g* for 10 min. The total protein content was determined using Bio-Rad protein assay reagent (Bio-Rad Laboratories, Hercules, CA, USA). Protein samples (40 μg) from each group underwent size fractionation via SDS–PAGE and were subsequently transferred onto a polyvinylidene difluoride membrane (Schleicher & Schuell, Middlesex, UK). Subsequently, the membrane underwent blocking with 5% skim milk in 10 mM Tris–HCl, containing 150 mM NaCl and 0.5% Tween 20 (TBS-T), followed by an overnight incubation at 4 °C with diverse primary antibodies specific to ERK, p-ERK, JNK, p-JNK, AKT, p-AKT, nuclear factor erythroid 2-related factor 2 (Nrf2), and MSP (Cell Signaling Technology, MA, USA). Following thorough washing with TBS-T, the membranes were subsequently exposed to horseradish peroxidase-conjugated secondary antibodies for a duration of one hour. Finally, the blots were visualized using an enhanced chemiluminescence detection system (Amersham, Piscataway, NJ, USA). 

### 4.11. Statistical Analysis

All statistical calculations were performed using GraphPad Prism software (version 6.0; GraphPad Software Inc., San Diego, CA, USA). All values are expressed as the mean ± standard errors of the means. Variations among the experimental groups were assessed through one-way analysis of variance (ANOVA), followed by post hoc Tukey–Kramer multiple comparison tests. Statistical significance was established at *p* < 0.05 for all analyses.

## Data Availability

Data is contained within the article.
